# A functional approach to the structural complexity of coral assemblages based on colony morphological features

**DOI:** 10.1038/s41598-017-10334-w

**Published:** 2017-08-29

**Authors:** Vianney Denis, Lauriane Ribas-Deulofeu, Nicolas Sturaro, Chao-Yang Kuo, Chaolun Allen Chen

**Affiliations:** 1 Institute of Oceanography, National Taiwan University, Taipei, 106 Taiwan; 2 Biodiversity Research Center, Academia Sinica, Nangang, Taipei, 11529 Taiwan; 3 Department of Life Science, National Taiwan Normal University, Taipei, 11677 Taiwan; 4 Biodiversity Program, Taiwan International Graduate Program, Academia Sinica and National Taiwan Normal University, Taipei, 11529 Taiwan; 5 ARC Centre of Excellence for Coral Reef Studies, James Cook University, Townsville, QLD, 4811 Australia

## Abstract

Colony morphological features is among the best predictor of the scleractinian coral’s function in reef ecosystems. However, morphological traits are categorical and to convert this information into a quantitative value as well as estimate their influence on ecosystem process remain a challenge. Here, we propose a trait-based approach to quantify morphological diversity and assess the structural complexity of the habitat provided by corals. We used a previously published dataset that is related to a bleaching event that affected the coral reef off Tikus Island in Indonesia in 1983. We found clear signs of recovery of the coral assemblage’s complexity toward pre El Niño conditions five years after the event. Independent of the change observed in species richness, this return in structural complexity was accompanied by a global decrease in species number associated with each particular morphological entity (Functional Redundancy) and an increase in the number of single-species entities (Functional Vulnerability). Together with species loss, we show an overall functional erosion of the coral assemblage and suggest that the role of the coral reef habitat could be strongly imperiled under repeated or synergistic disturbances. This approach offers an opportunity for a better understanding of coral responses to natural and anthropogenic disturbances.

## Introduction

The structural complexity of a coral reef is hypothesized to promote species richness and abundance^1^ by mediating critical ecological processes such as recruitment, recovery, predation, and competition (*e*.*g*. refs 2–5). Over the last few decades, coral reef communities have been rapidly reconfigured under increasing natural and anthropogenic disturbances^6^. Mechanical damage to corals induced by typhoons as well as reduced growth rates following mass mortality caused by bleaching events have engendered a major decrease in the structural complexity of coral reefs^7^. This trajectory has often been associated with a loss of coral cover to the benefit of non-calcified or opportunistic organisms (such as some macro-algae), together with a degradation of the coral reef habitat^8^. This simplification of benthic assemblages, characterized by a loss of structural complexity, is today jeopardizing the functions of this ecosystem and thus its ability to support high diversity^9^.

Coral reef rugosity (*i*.*e*., variations of amplitude in the height of a surface)^10^ has traditionally been used as a proxy for structural complexity^11, 12^, and the loss of rugosity is associated with reef degradation^13, 14^. This is the key parameter, aside from water depth, for predicting the trajectory of coral reef ecosystems after disturbances^5^. Through their impacts on reef accretion, ecological shifts in the Caribbean have been associated with flattening coral reefs^7^ and substantial changes in reef rugosity^15^. In addition, Bozec *et al*.^16^ predicted a dramatic loss of Caribbean reef rugosity in the coming decades due to severe and frequent bleaching events. Although reefs with high rugosity and high micro-topography typically provide more protection and ecological niche space for reef fish and sessile invertebrates to settle^1, 17–19^, rugosity is a poor indicator of the actual shape of the reef, which determines the ‘quality’ of the habitat structure^16^.

Traits measure species role and performance, and help in understanding the fundamental and realized niche processes of the species present in a community^20^. For corals, the trait-based approach has been applied to define adaptive strategies and the responses of the assemblage to disturbances^21, 22^. Consistent with ecological shifts observed in coral reefs, four strategies based on a panel of coral traits have emerged to predict the impacts of environmental and anthropogenic stressors on species assemblages: competitive, weedy, stress-tolerant, and generalist^22^. Competitors have been hypothesized to be the most sensitive to bleaching, which could lead to their future declines/dimiss^22, 23^. However, other ecological responses may exist as observed on Scott Reef in Australia, where competitors have recovered within 12 years of a coral bleaching event^24^. Although traits associated with species performance (*i*.*e*., individual responses and “functioning” traits) can vary among locations and over time, characteristics that are associated with species’ roles (*i*.*e*., species interaction and “functional” traits) are less plastic and more stable over time. The latter contribute to the structurally complex habitats characteristic of reefs^1^ and to the production of biomass that enters the food web^25^.

Instead of rugosity, an alternative way to reveal structural complexity is to look at coral colony morphology. Morphological diversity is the foundation of a complex habitat and it promotes species interactions^26^. In addition, morphology is the primary determinant of adaptive strategies^22, 27^ and is among the best predictors of coral assemblage responses to disturbance^22, 28^. Morphology also conditions structural complexity^29^, which plays a central role in ecological recovery^5^ – through its influence on growth rates^30, 31^ and demographic processes^30^. Recently, colony morphological features have been proposed as a basis for inferring unmeasured growth rates and a key component of a coral supertrait^32^, as they can encompass large variations in a broad range of biological, ecological, and evolutionary processes^32^. Therefore, morphology could be among the most relevant indicator (and integrator) of reef conditions and a major determinant of reef functioning: *e*.*g*., the coral’s ability to maintain a functional habitat.

Because of the roles they play in ecosystem processes and their overall contribution to coral reef resilience, morphological groups and species richness have traditionally been used to compare functional compositions of coral assemblage^6^. Furthermore, coral species are easily categorized morphologically. On Caribbean reefs, morpho-functional groups are characterized by a lower number of species (lower redundancy) than in the Indo-Pacific; therefore, Caribbean reefs have been hypothesized to be more vulnerable and less resilient to environmental degradation^6^. Combined with information on cover, the relative contributions of different coral morphologies of high-latitude coral assemblages exhibit reduced functionality characterized by particularly high redundancy in a single functional group^33^. Similarly, typhoons have only minor effects on the morpho-functional composition of an Okinawan mesophotic coral assemblage despite profound reductions in coral cover^34^. Therefore, colony morphological features could be a critical parameter for estimating structural complexity.

To date, studies that have evaluated morphological-group richness have remained particularly descriptive and qualitative. Here, we propose a quantitative approach which derives directly from field survey data and offers an easy way to assess and monitor the Functional Diversity (*FD*) - the range of things that organisms do in communities and ecosystems^35^ - of coral assemblages based on simple traits; *i*.*e*., colony morphologies. We based our approach on the evolution of the morpho-functional space of a coral assemblage reflecting the modification of the structural complexity (‘quality’) of the coral habitat. We used a dataset describing the effects of a bleaching episode, triggered by the 1983 El Niño event on the coral assemblage at South Tikus Island, Indonesia, and its subsequent recovery^36, 37^.

We applied different sets of functional indices reflecting the multiple facets of *FD* and which have emerged as important indicators for documenting the complex nature of change in disturbed ecosystems. We eventually summarized our results in terms of the effects of an isolated disturbance (such as coral bleaching) on the Functional Redundancy (number of species performing similar functions), Over-Redundancy (overrepresentation of some functions in terms of species richness) and Vulnerability (number of functions supported by only one species) of a coral assemblage. These results are then discussed in the context of recurrent bleaching events and synergistic disturbances. The application of this functional approach in a historical context will open a new window on ongoing changes affecting coral reefs worldwide.

## Materials and Methods

### Dataset

To illustrate our approach, we used a dataset documenting the effects of a bleaching event (triggered by the 1982/83 El Niño Southern oscillation event) occurring in 1983 on the coral assemblage at Tikus Island, south of the Thousand Islands Archipelago, Indonesia, and subsequent changes over five years. The data consist of the percent cover of 75 coral species measured along ten 30 m line transects laid perpendicularly to a main transect positioned permanently across the reef flat. The first survey was conducted before the bleaching event in 1981, and surveys were repeated in 1983, 1984, 1985, 1987, and 1988. The differences in transect position were estimated to be no greater than 0.5 m in subsequent years. Further information on the sampling sites and methods can be found in Brown & Suharsono^38^ and Warwick *et al*.^36^. The dataset (coral cover) is also accessible from the R package ‘mvabund’^39^, which provides tools for the model-based analysis of multivariate abundance data in ecology.

### Coral morpho-functional traits

According to earlier studies addressing the ecological roles of corals^6, 33^ and our own experience, eight ‘typical’ morphologies (traits) were considered to contribute to the structural complexity of reefs: arborescent, bushy, table, foliose, column, massive, encrusting, and unattached. We classified all coral species into these eight distinct traits. These morphological traits were chosen to limit ambiguity and also to capture a range of shapes that we hypothesize might relate to the functional role of the species in the ecosystem (see also^6, 27, 33^). Morphological traits were considered as unique binary traits and each species was assigned to one or more of these categories (using 0 and 1). This method incorporated growth form plasticity (see Supplementary Table S1). Free-living Fungiidae were consistently categorized as both unattached and massive, traits they tend to follow as they grow larger.

### Functional space of morphological diversity

A dissimilarity matrix was computed among species using Gower distance^40^, which can handle a variety of data types, including binary. A Principal Coordinates Analysis (*PCoA*) was performed on this functional matrix using the Cailliez correction to accommodate negative eigenvalues^41^. Morpho-functional entity coordinates on the first two principal axes were used to build a multidimensional functional space, which illustrates the relationships among species and incorporates species morphological plasticity. The eight traits (typical morphologies) were overlaid as vectors to easily discriminate the sources of the differences among species.

### Functional indices

In addition to species richness (*N*) and coral cover, a variety of indices reflecting the multiple facets of *FD* were used to describe potential changes in the coral assemblage. The total number of Functional Entities (*FE*) was defined as unique combination of the eight morphological traits. Singular function integrates potential morphological plasticity within a species. The distance between *FE* on the functional space reflects the degree of morphological difference. *FE* was computed for each year and transect. In addition, we reported the average *FE* number by transect ($\overline{FE}$) and the ratio $\overline{FE}/FE$, which is an indicator of the regularity (degree of homogeneity) in the distribution of the *FE* among the transects. This ratio (between 0 and 1) illustrates the proportion of shared *FE* among transects.

Community-level Weighted Means (*CWM*, the percent contribution of a given trait to the coral assemblage), were calculated for each of the eight traits. Functional Richness (*FRic*) represents the volume inside the convex hull shaping all the *FEs* on the morpho-functional space. Functional Evenness (*FEve*) measures the regularity of the abundance distribution in the functional space. Functional Divergence (*FDiv*) reflects the proportion of the total abundance that is supported by the species with the most extreme functions. Functional Dispersion (*FDis*) represents the abundance-weighted deviation of species trait values from the center of the functional space filled by the community (*i*.*e*., the abundance-weighted mean distance to the abundance-weighted mean trait values of the community). Finally, Functional Entropy (Rao Index, *Q*) characterizes the abundance-weighted sum of pairwise functional distances between species. Further details of these indices, their definitions, their uses, and their ecological relevance are reviewed in Mouillot *et al*.^42^.

The indices were calculated for each transect and summarized by year as mean ± standard error. Transect data have previously been considered as dependent samples over the years^38^. Indeed, consistent changes in the diversity and structure of the coral assemblage were observed on the transects; however, following a more conservative approach, Warwick *et al*.^36^ treated transects within a year as non-blocked replicates. Here, we considered transects as independent replicates over the years. The effects of coral bleaching on *FD* and subsequent recovery were analyzed using a two-tailed Student’s *t*-test. Welch correction was used to take into account unequal variances between the samples. To account for false positive, P-values were adjusted for multiple comparisons^43^. However, the P-values from two-tailed paired *t*-tests were also reported as they could be of relevance in interpreting early changes in benthic assemblages. Paired Student’s *t*-tests were performed between years only for transect where the computation of the functional indices was possible (<3 functionally singular species on a transect).

With *N* as the total number of species in a year, *FE* as the total number of Functional Entities, and *n*_*i*_ as the number of species in the *FEs*, we calculated Functional Redundancy (*FR*), Functional Vulnerability, and Functional Over-Redundancy for every year. Following Mouillot *et al*.^44^, the indices were expressed as:

1 $${\rm{Functional}}\,{\rm{Redundancy}}=\frac{{\sum }_{i=1}^{FE}{n}_{i}}{FE}=\frac{N}{FE}$$

2 $${\rm{Functional}}\,{\rm{Vulnerability}}=\frac{FE-{\sum }_{i=1}^{FE}\min ({n}_{i}-1,1)}{FE}$$

3 $${\rm{Functional}}\,\mathrm{Over} \mbox{-} \mathrm{Redundancy}=\frac{{\sum }_{i=1}^{FE}[\max ({n}_{i},FR)-FR]}{S}$$

To assess whether the observed values of Functional Vulnerability and Functional Over-Redundancy were higher than expected by chance, we tested them under a null model in which species were randomly assigned to *FEs* following Mouillot *et al*.^44^. Briefly, we simulated 9,999 assemblages for each year, ensuring that each *FE* had at least one species. Functional Vulnerability and Functional Over-Redundancy, were calculated by keeping *N* and *FE* constant. The Standardized Effect Size and P-value (quantile) were used to compare the observed and null assemblages. Eventually, relationships between *FE*, $\overline{FE}$, $\overline{FE}/FE$, Functional Redundancy, Functional Vulnerability, Functional Over-Redundancy, and *S* were tested using Pearson correlations. A list of abbreviations and their respective definitions is provided in Table 1.

**Table 1:** List of abbreviations and their respective definitions.

Abbreviation	Definition
*CC*	Coral Cover
*CWM*	Community-level Weighted Means
*FD*	Functional Diversity
*FDis*	Functional Dispersion
*FDiv*	Functional Divergence
*FE*	Functional Entities
$\overline{FE}$	Average *FE* number by transect
*FEve*	Functional Evenness
*FRic*	Functional Richness
*N*	Species richness
*n*_*i*_	Number of species in the FEs
*PCoA*	Principal Coordinates Analysis
*Q*	Rao Index
*SES*	Standardized Effect Size

All of the analyses were performed in R (v3.1.1)^45^ using the packages ‘cluster’^46^, ‘fpc’^47^, ‘plotrix’^48^, and ‘FD’^49, 50^ for computing functional diversity indices.

### Data availability

The South Tikus Island data set is available from the R package ‘mvabund’^39^.

## Results

**Figure 1:**
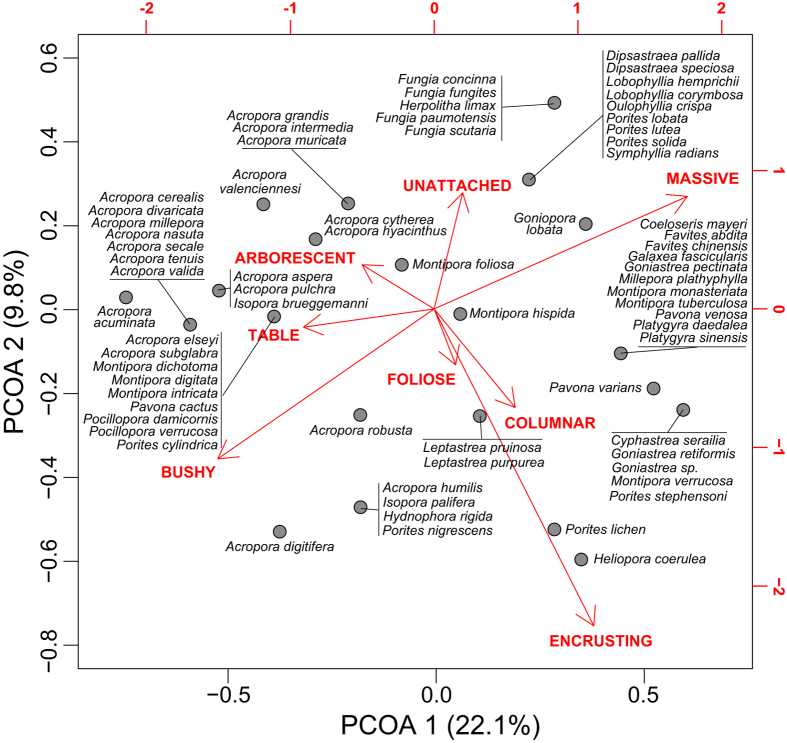
Morpho-functional space. The distribution of Functional Entities (group of species based on unique combinations of morphological traits) is shown in a functional space where *PCOA1* and *PCOA2* represent axes extracted from a Principal Coordinate Analysis (*PCoA*) on the morphological features of coral species. Morphological traits were overlaid as vectors on the *PCoA* to easily discriminate sources of the differences among species. *PCoA* was built on a Gower distance dissimilarity matrix. Cailliez correction was applied to accommodate negative eigenvalues.

**Figure 2:**
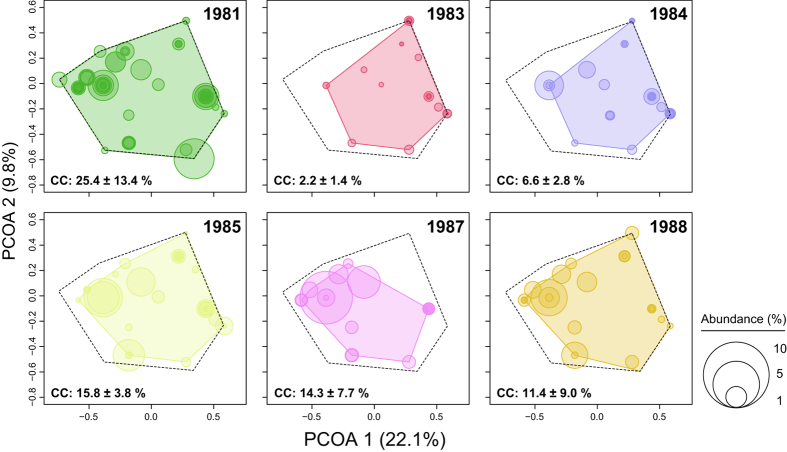
Evolution of morpho-functional space from 1981 to 1988. Circles represent species occurrence in the coral assemblage and are proportional to species relative abundance (%). Colored polygons characterize functional space filled by species (*i*.*e*., Functional Richness, *FRic*). Pre-bleaching *FRic* is symbolized by a dash line and the change in *FRic* displayed as the difference from the convex surface of the *FRic* for the following years. Coral Cover (*CC*) is given as mean ± standard error.

The categorization of the 75 coral species according to the eight traits resulted in 21 *FEs*, which represents 8% of the theoretical number (256) of unique combinations (Fig. 1). The convex hull shape and the species relative contribution to the FEs reflects the major changes that occurred in the coral assemblage over the years (Fig. 2). In 1983, the morpho-functional space clearly shows the loss of branching species (bushy, table, arborescent traits). In 1988, the reduced relative contributions of corals presenting massive, columnar, and encrusting traits are particularly obvious, while the general shape of the convex surface tends to come back to the initial shape.

**Figure 3:**
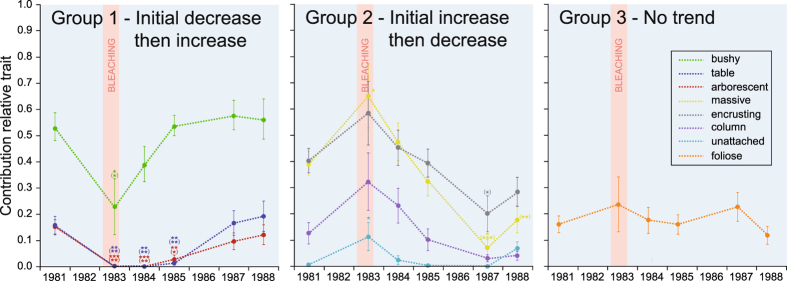
Evolution of the Community-level Weighted Means (*CWM*) of morphological traits from 1981 to 1988. Three groups of morphological traits were distinguished according to their response to disturbance: traits that showed a sharp decrease in their contributions to the assemblage after bleaching and then progressive recoveries (Group 1), traits that showed initial increases in their contributions and then progressive decreases (Group 2), and traits with no trends identified (Group 3). The bleaching event (mean seawater temperatures + 2–3 °C over a six-month period^38^) is symbolized by the red area. Error bars are standard errors. Levels of significance of the *t*-tests: ***<0.001, **<0.01, *<0.05. Asterisks in parentheses indicate levels of significance for paired *t*-tests.

Three groups of morphological traits were identified in the *CWM* analysis according to their response to bleaching (Fig. 3). Bushy, table, and arborescent traits (Group 1) decrease significantly in their contributions after a bleaching event before recovering to initial levels within six years. Massive, encrusting, columnar, and unattached traits (Group 2) initially increased (significantly for massive and unattached) before progressively decreasing until 1987, when their contributions were lower than their original contributions (significantly for massive and encrusting). In 1988, their relative contributions were still lower than their initial contributions (significantly for massive), except for the unattached trait, which had a slightly higher relative contribution than in 1981. The relative contribution of the foliose trait (Group 3) was stable over time. The yearly depiction of *CWM* (Supplementary Fig. S1) shows a clear recovery toward the initial contribution of morphological traits in the coral assemblage, but a depletion in encrusting, columnar, and especially massive corals remained obvious in 1988.

**Figure 4:**
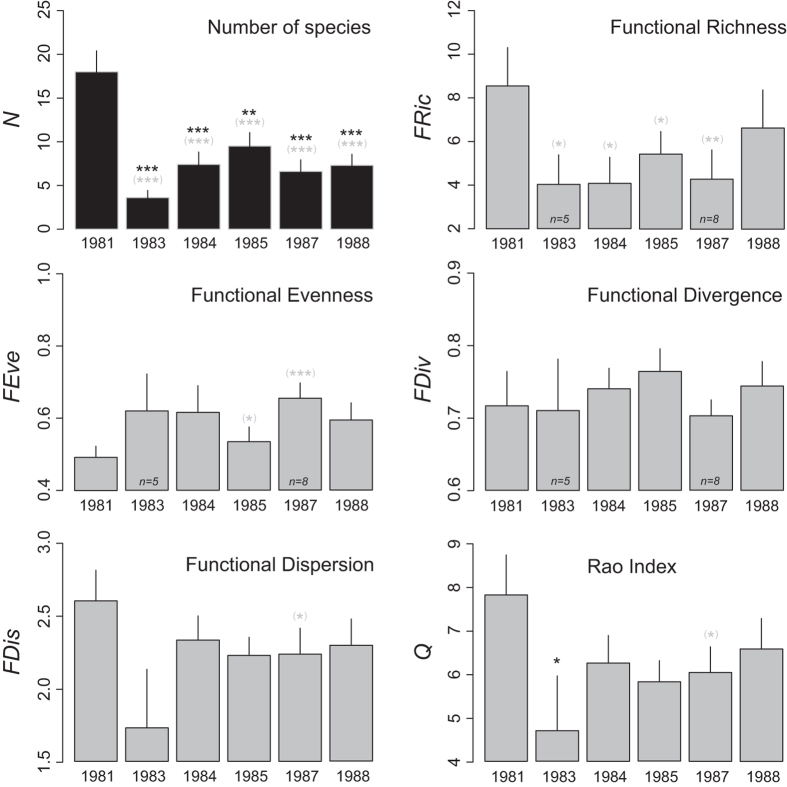
Evolution of the average species richness and functional diversity from 1981 to 1988. Histograms show the number of species (*N*), Functional Richness (*FRic*), Functional Evenness (*FEve*), Functional Divergence (*FDiv*), Functional Dispersion (*FDis*), and Rao’s quadratic entropy (*Q*) indices. Error bars are standard errors with n = 10 except where indicated in the bars. Levels of significance of the *t*-tests are symbolized by black asterisks: ***<0.001, ** < 0.01, *<0.05. Grey asterisks in parentheses indicate levels of significance from paired *t*-tests.

The total number of species at the study site dropped from 54 in 1981 to 21 in 1983 after the bleaching event (Table 2). Species number increased from then until 1985 (33 species) before decreasing again to its lowest level in 1987 (18 species). In 1988, species richness at the study site (25 species) had not recovered to its original level. Similarly, the lowest average number of species identified on transects in 1983 occurred immediately after the bleaching event (Fig. 4). It increased until 1985 before another decrease occurred in 1987, which was eventually followed by a slight rise in 1988.

**Table 2:** Total number of species, Functional Entities, Functional Redundancy, Functional Vulnerability, and Functional Over-Redundancy by year.

	1981	1983	1984	1985	1987	1988
**Total number species** ***N***	54	21	29	33	18	25
**Functional Entities** ***FE***	19	11	11	16	10	15
Average *FE*/transect $\overline{FE}$	9.70 ± 0.83	2.80 ± 0.51	5.00 ± 0.77	6.50 ± 0.78	5.30 ± 0.85	6.00 ± 1.05
Ratio $\overline{FE}/FE$	0.51	0.25	0.45	0.40	0.53	0.40
**Functional Redundancy**	2.84	1.90	2.64	2.06	1.80	1.67
**Functional Vulnerability**	47%	55%	45%	63%	60%	67%
	vs. 15 ± 7%	vs. 39 ± 9%	vs. 18 ± 9%	vs. 33 ± 8%	vs. 43 ± 9%	vs. 50 ± 7%
	*SES*: 4.77	*SES*: 1.74	*SES*: 2.97	*SES*: 3.64	*SES*: 1.79	*SES*: 2.35
	***	n.s.	**	***	n.s.	*
**Functional Over-Redundancy**	35%	26%	30%	33%	27%	27%
	vs. 18 ± 3%	vs. 18 ± 4%	vs. 19 ± 4%	vs. 18 ± 4%	vs. 19 ± 4%	vs. 20 ± 3%
	*SES*: 4.29	*SES*: 1.74	*SES*: 2.07	*SES*: 2.81	*SES*: 1.79	*SES*: 2.35
	***	n.s.	**	***	n.s.	*

Functional Vulnerability and Functional Over-Redundancy values are given versus values from null models with 9,999 permutations. Mean value are given ± standard error. *SES*: Standardized Effect Size. Levels of significance: ***<0.001, **<0.01, *<0.05. n.s.: non-significant.

*FRic*, and *Q* collapsed after the bleaching event between 1981 and 1983. The paired Student’s *t*-tests suggest that *FRic* did not recover to its initial level before 1988. *Q* recovered to its pre-El Niño level in 1984, decreased again in 1987, and recovered to its initial level in 1988.

Despite being not significant, *FDis* also presented a decrease in 1983 which seemed to have recovered the year after in 1984. *FDis* significantly decreased in 1987, before to get back its initial level in 1988. *FEve* followed a different pattern, with the paired Student’s *t*-tests revealing an increase in 1985 and 1987. In 1988, *FEve* regained its initial level. To contrast, *FDiv* was relatively stable over time without any significant changes in its level over the 1981–1988 time period.

After an initial decrease caused by coral bleaching, *FE* and $\overline{FE}$ tended to recover to their initial levels (Table 2), but there was a hiatus in their recovery in 1987. Of the 19 FEs that were recorded at the study site in 1981, only four of them were not in the 1988 coral assemblage. The reduced $\overline{FE}/FE$ ratio suggests that the *FE* distribution at the study site became more heterogeneous following the disturbance. This remained below its original level in 1988, showing that coral bleaching could have a long term effect on the frequency of *FE* observed on the transects.

The number of species in the *FEs* changed over the time (Supplementary Fig. S2). Functional Redundancy was negatively affected by the bleaching event. After a period of recovery between 1983 and 1984, Functional Redundancy decreased continuously until 1988. Before the disturbance, about half of the *FE* (47%) was supported by a single species and 35% of the species contributed to Functional Over-Redundancy. Functional Vulnerability increased from 47% to 55% after the bleaching event before decreasing to below its initial level (45%) in 1984. In subsequent years, Functional Vulnerability increased and eventually reached 67% in 1988. Functional Over-Redundancy was less affected by the El Niño event, decreasing to 26% after the bleaching event, recovering to 33% in 1985, and then further sinking to 27% in 1988. $\overline{FE}/FE$ (R^2^ = 0.13, p = 0.48), Functional Redundancy (R^2^ = 0.64, p = 0.06), and Functional Vulnerability (R^2^ = 0.24, p = 0.33) were not correlated with variations in species richness. In contrast, *FE* (R^2^ = 0.75, p < 0.05), $\overline{FE}$ (R^2^ = 0.77, p < 0.05), and Functional Over-Redundancy (R^2^ = 0.83, p < 0.05) were positively correlated with the total number of species observed every year. Most of the Functional Over-Redundancy values were significantly higher than expected by chance (Table 2) except in 1983 and 1987, suggesting that the species were not packed randomly into a few *FEs*.

## Discussion

Coral morphologies always turn up as major determinants of ecosystem functioning given their importance in differentiating adaptive strategies^22^, predicting the responses of coral assemblages to disturbances^22, 28^, and conditioning structural complexity^29^ - the latter having a central role in shaping biological communities^1, 29^ and their ecological recovery^5^. Here, we analyzed the impact of an isolated disturbance (*i*.*e*., bleaching event) on the morphological (functional) diversity of the fringing reef flat of South Tikus Island located in Indonesia.

We describe clear signs of the return of the coral assemblage’s functionality toward the pre El Niño state. However, this partial recovery is accompanied by a decrease in Functional Redundancy together with an increase in Functional Vulnerability. Overall, our results demonstrate a functional erosion of a coral assemblage and suggest that the role of coral reefs could be strongly imperiled under repeated or synergetic disturbances.

South Tikus reef was severely affected by the bleaching event, and five years later its coral cover and species richness were still less than half of their original values. Despite these significant declines, there were no significant differences between 1981 and 1988 in coral diversity and evenness indices^38^, nor in dominance patterns^36^. Multivariate analyses of the coral assemblage revealed that the community was recovering to its pre-El Niño state, but by 1988 this process was still incomplete^36^. This period was marked by a hiatus in recovery between 1985 and 1987. Anderson *et al*.^37^ demonstrated that changes in community structure were mainly affected by changes in species richness. Similarly, we observed that the number of *FEs* (Functional Entities) in this coral assemblage was related to the number of species recorded. *FRic* (Functional Richness) and species richness are generally positively correlated^51^ since communities with more species are more likely to contain a higher diversity of traits and thus perform more functions^52^.

In total, 21 *FEs* were identified based on the classification of 75 coral species composing the benthic assemblage at the study site. Roughly half of them (48%) were supported by only one species, highlighting the unique and irreplaceable habitat offered by some coral taxa. In tropical fish communities, it has been shown that some of those species may play critical roles in ecological processes and thus underpin resilience through their functions in coral reef recovery after disturbance^53–55^. Of the 19 *FEs* present in 1981, 11 (58%, 21 species) persisted into 1983 after the bleaching event. Modifications in the coral assemblage were characteristic of a niche-based change^42^, with an almost complete loss of the left part of the functional space that *CWM* (Community-level Weighted Means) indicated corresponds to the decline in all branching species (*CWM* – Group 1), with Group 2 taking over their contribution to the coral assemblage. In particular, *Acropora*, *Pocillopora*, and *Montipora* were affected by the disturbance^38^, which is consistent with the general agreement that branching and fast-growing corals are more sensitive to temperature anomalies than massive and encrusting slow-growing corals^56, 57^. This finding is consistent with massive and encrusting species surviving the 1998 bleaching event in Kenya^23^. There, reefs dominated by slow-growing and ‘stress-tolerant’ corals saw these species performing better in response to the thermal stress and then dominate the coral assemblage in subsequent years^23^. After 1985, *FEs* at Tikus recovered with functional space being progressively recolonized toward the pre-El Niño conditions. An interruption in this recovery occurred in 1987, which, in terms of species richness, was associated with an unknown disturbance that affected the reef ^36^. This was characterized by a decrease in the contribution of *CWM* Group 2 species (top-right part of the functional space), suggesting that the disturbance originated from a different cause than the 1982/83 situation. However, the decrease observed in 1987 seems to have been initiated years before in 1984 following the bleaching event, and could be a long-term consequence of this episode. The exceptional recovery of branching corals was mainly driven by the capacity of *Acropora* species to restore their population. *Acropora* species were indeed decimated in 1982/83, and not a single colony appeared to survive the bleaching event. They reappeared on transects in 1985, and regained their original functional contribution to the coral assemblage by 1988. The remarkable self-regeneration ability, termed the ‘Phoenix effect’^58^, could be explained by remnant cryptic patches of tissue surviving the bleaching event. In this way, *Acropora* populations can restore decimated populations in <1 year^59^. Alternatively, recruitment from unaffected deeper (‘deep reef refugia hypothesis’^60^) and/or distant^61^ reefs could have contributed to the recovery. However, this process offers a slower trajectory toward the recovery of coral populations^59^, with the exception of brooders, in which life traits^62^ could considerably shorten the time needed^63, 64^. *Acropora* species are typically spawners and usually do not present this characteristic of ‘weedy’ species. Therefore, recruitment could have a minimum role in the pattern observed here in comparison to the self-regeneration of coral colonies. A similar trajectory has been observed in Australia at Scott Reef after the 1998 bleaching event where, with a negligible supply of larvae from outside the reef, coral assemblages dominated by Acroporidae species mostly recovered by 2010^24^. In this context, *Acropora* species (dominating Group 1) could be the short term losers but the long term winners^65^ after coral bleaching. The branching species *Montipora digitata* also strongly affected the dynamics of our functional space. This coral was a dominant species in 1981, but declined in 1983 before recovering in 1984 and thrived in subsequent years. In 1987, this species occupied 44% of the total live coral cover on transects^38^. Because of its morphological plasticity, *Heliopora coerulea* plays a unique role in the coral assemblage, together with *Porites lichen* explaining one part of our functional space. This was an abundant species prior to 1983, but it disappeared from transects after the bleaching event. It only reappeared in 1988, at very low levels of coverage, offering only an incidental contribution toward its function. Some *H*. *coerulea* colonies were removed from the reef flat for the construction of a small jetty during 1981–1983^38^; therefore, the demise of this part of the functional space cannot be solely attributed to seawater warming. In 1995, thirteen years after the bleaching event, coral cover remained below the 1988 level at 19.2%^21^. Since then, coral cover seemed relatively stable as it was recorded at similar levels during a survey on coral diseases across the Thousand Islands Archipelago in 2011–2012^66, 67^. On this reef, now chronically affected by pollution from the nearby Jakarta Bay^68^, *Montipora* ^66^ and corals characterized by massive trait^21^ have become the dominant features of the coral assemblage. Coral diseases are highly prevalent on this reef^66^, which has a high abundance of algae^21, 66^. Successive bleaching events on this mid-shore reef (after the 1982/1983 severe bleaching event) have been proposed to have dismissed most of remnant branching and unattached (free-living) coral entities in the assemblage^21^. The gradual removal of bleaching susceptible individuals, notably during the 1997/1998 El Niño event, has been suggested as modulating the response to the 2010 bleaching event across the Java Sea^69^. Coral mortality, especially in branching taxa, was low in comparison to other locations with different thermal histories in Indonesia^69^. The 1997/1998 El Niño and 2010 bleaching events did not affect much the corals at Tikus, whereas the 2016 bleaching event was reported to impact only 5–10% of the corals (M. Abrar, personal communication). Eventually, the outcome of the long-term dynamic observed today at South Tikus is consistent with coral life-history strategies previously defined^22^. It results in a shift from ‘competitive’ fast-growing branching and plating species to ‘stress-tolerant’ slow-growing, long-lived massive, submassive, and encrusting species^23^.

In addition to the decrease in species richness, there were drastic changes in functional indices after the disturbance. *FRic*, which is the proportion of the functional space filled by *FE*, decreased by half after the disturbance mainly because of a loss of branching entities. *FRic* started to increase in 1985, but recovery was interrupted in 1987. In 1988, *FRic* recovered, but at lower levels from its original value. The unique functions performed by *Acropora acuminata*, *A*. *valenciennesi*, and *A*. *digitifera* were swept away by the bleaching event, as these species never reappeared in the coral assemblage. The loss of these species explains the main differences observed in the proportions of the functional space filled by the coral assemblage. *FDis* (Functional Dispersion), which reflects changes in the abundance-weighted deviation of species trait values of the community^42, 49^, decreased after the disturbance. This decrease was not significant and a fast recovery of *FDis* was observed as early as in 1984 with however a hiatus observed in 1985–1987, while the functional space was recolonized. Rao’s quadratic entropy *Q* has been suggested as being among the best solutions for synthetizing species trait information and abundance in order to measure *FD* (Functional Diversity)^70^. Together with its transformation, *Q* (Functional Entropy) has been extensively used in studies that have quantified *FD* ^71–73^. In our functional space, *Q* reflected the changes in the abundance-weighted sum of pairwise functional distances between species^42, 70^. *Q* followed a similar pattern as *FDis*; however, a sharper (and significant) decline was observed after the disturbance. Recovery is initiated in 1984, with a pronounced interruption in its recovery in 1985–1987. Mouillot *et al*.^42^ mentioned that indices based on species traits and abundance together could act as early-warning indicators. Our results suggest that the hiatus in the recovery of the coral assemblage^36^ could have been initiated as early as 1985. The bleaching event increased the regularity of the abundance distribution in the functional space (Functional Evenness, *FEve*).

The loss of a dominant branching functional group immediately after the bleaching event tended to homogenize the abundances of the remaining *FEs*. Their recovery, accompanied by further changes to the functional space mainly characterized by the loss of massive trait species, resulted in a significant increase of *FEve* along the 1985–1987 period. *FEve* was further reduced to the same level as prior to the disturbance in 1988. *FEve* typically decreases proportionally with disturbance intensity, whereas *FDiv* (Functional Divergence) usually exhibits a decreasing logarithmic relationship. In our study, apart from a slight decrease in 1987, *FDiv* was unaffected by the disturbance, which indicates that the proportion of the total abundance that was supported by species with the most extreme functions did not change over the years. It suggests that despite contrasted physiological response to coral bleaching^56^, range of habitat offered by the coral assemblage seems to have been preserved over the years. We hypothesize that the capacity to maintain high *FDiv* could be a critical characteristic supporting the recovery of coral assemblages. It could encourage the diversification of functional niches and favor key organisms that are involved in ecosystem resilience.

Despite a significant recovery toward its original functions, the 1988 coral assemblage had an increased susceptibility to disturbance. Correlated with species loss, the number of *FEs* observed and the frequency of their observations on transects ($\overline{FE}/FE$) decreased after the bleaching event. Before the disturbance, species tended to be packed into a few specific *FEs*, while leaving almost half of the *FEs* (47%) highly vulnerable. The disturbance resulted in a decrease in Functional Redundancy, which was characterized by a decrease in the over-representation of species in a few *FEs* and an increase in the number of *FEs* supported only by a single species. In 1988, and independent of species richness, we observed a sharp increase in the Functional Vulnerability of the coral assemblage, which was accompanied by a decrease in the Functional Redundancy and Functional Over-Redundancy. These results demonstrate a functional erosion of the coral assemblage occurring after a bleaching event and suggest that the role of the coral reef could be strongly imperiled in the future under repeated or synergistic disturbances (Supplementary Video 1). In light of the recent bleaching events affecting Indo-Pacific reefs, it would have been interesting to evaluate the implications of our results on the current response of coral assemblages. Recent study at the shallow reef flat at Bunaken (North Sulawesi, Indonesia) showed that coral mortality was not solely induced by the 2015–2016 El Niño event, but also by a rapid sea level fall observed on that year^74^. It caused up to 85% mortality on reefs dominated by massive and encrusting corals such as *Porites*, *Goniastrea*, *and Heliopora* ^74^.

Unfortunately, work at Tikus Island has not been followed up with the exception of punctual surveys published (using different methodology) on the environmental conditions across the Thousand Islands Archipelago in 1995^21, 75^, 2005^68^, and 2011/2012^66, 67, 76^. All showed a dominance by massive and encrusting coral taxa on reefs surrounding South Tikus. In addition, an analysis of the coral diversity revealed that of the 75 species recorded across the 1981–88 period at South Tikus, 17 were not observed anywhere on the archipelago in 2005^77^. This clear decline in species number has been interpreted as a consequences of a general degradation in the coral reefs caused by the synergetic effects of natural and anthropogenic disturbances^77^, which could have consequences on the entire reef communities^68^. Overall, it highlights the importance of maintaining long-term surveys to better interpret current and future responses of coral assemblages to climate changes.

In conclusion, this functional approach provides a qualitative and quantitative assessment of the habitats provided by corals. Information regarding morphological traits can easily be obtained from the field and the approach integrates the plasticity of coral colony morphology. The identification of coral species or knowledge of their physiology is not required. If combined with rugosity measurements, this approach would accurately characterize the structural complexity of reefs, which has been identified as a critical facet of their recovery potential (and by extension their resilience^78^). The relationship between phylogenetic diversity and *FD* should be investigated in future studies to ascertain whether phylogenetic information can provide relevant and similar information regarding changes in the roles of coral assemblages in response to disturbance. Furthermore, a generalization of this approach would facilitate the identification of various facets of coral reef degradation and functional recovery. The potential extension of this approach to other benthic organisms would further contribute to our understanding of the roles of the coral reef and expected changes under increasing disturbances.

## Electronic supplementary material

Supplementary Table and Figures

Supplementary Video 1
