# Supplementary material for: A functional approach to the structural complexity of coral assemblages based on colony morphological features
Source: Sci Rep. 2017 Aug 29;7:9849. doi: 10.1038/s41598-017-10334-w (PMC5575326; doi:10.1038/s41598-017-10334-w)
Supplement: Supplementary file 1 — Supplementary Table and Figures [file 41598_2017_10334_MOESM1_ESM.doc]

**A functional approach to the structural complexity of coral assemblages based on colony morphological features**

Vianney DENIS, Lauriane RIBAS-DEULOFEU, Nicolas STURARO, Chao-Yang KUO, Chaolun Allen CHEN

**Supplementary Table S1. Categorization of coral species according to eight morphological traits.**

| **Species** | **Arborescent** | **Bushy** | **Table** | **Columnar** | **Massive** | **Encrusting** | **Unattached** | **Foliose** |
| --- | --- | --- | --- | --- | --- | --- | --- | --- |
|  | 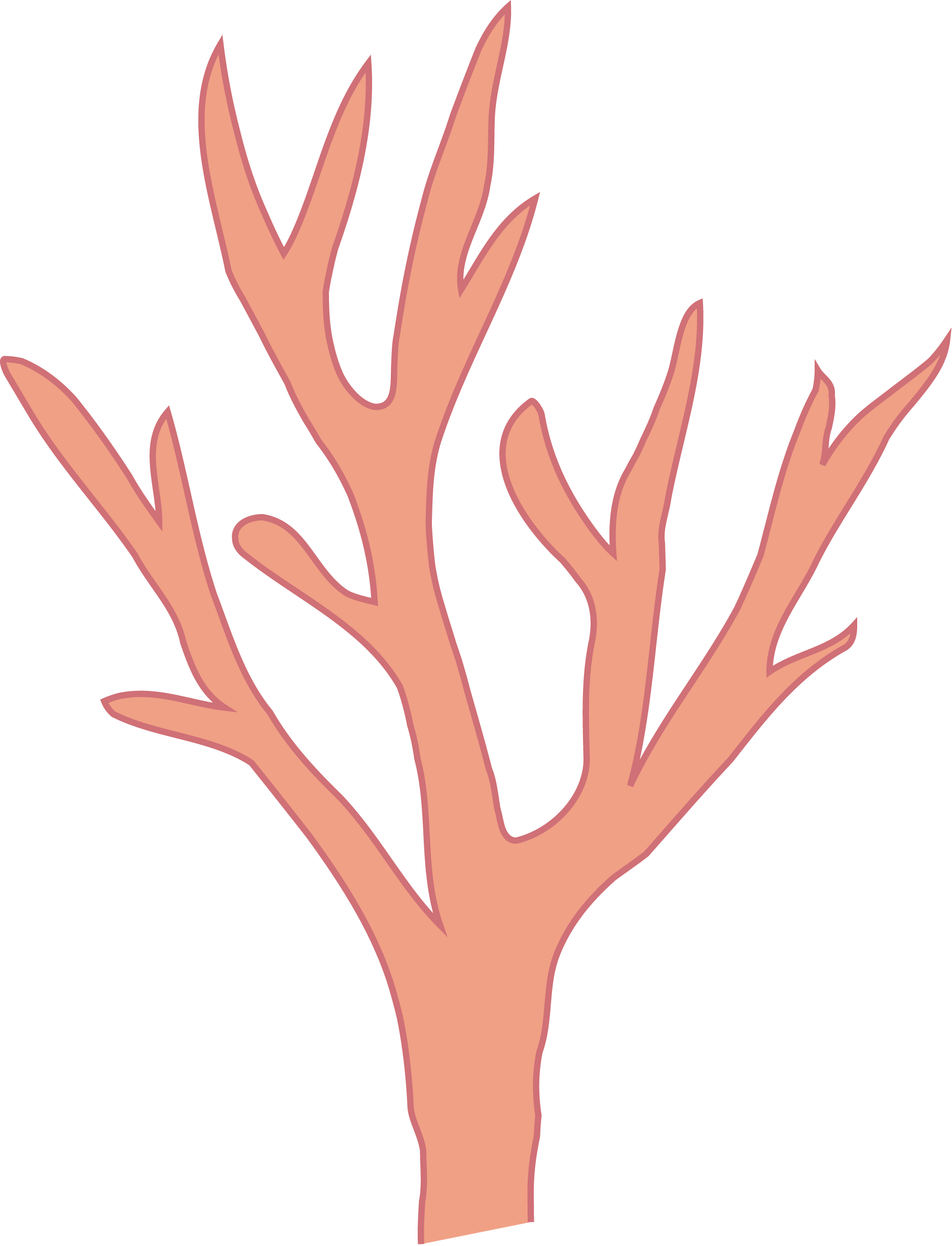 | 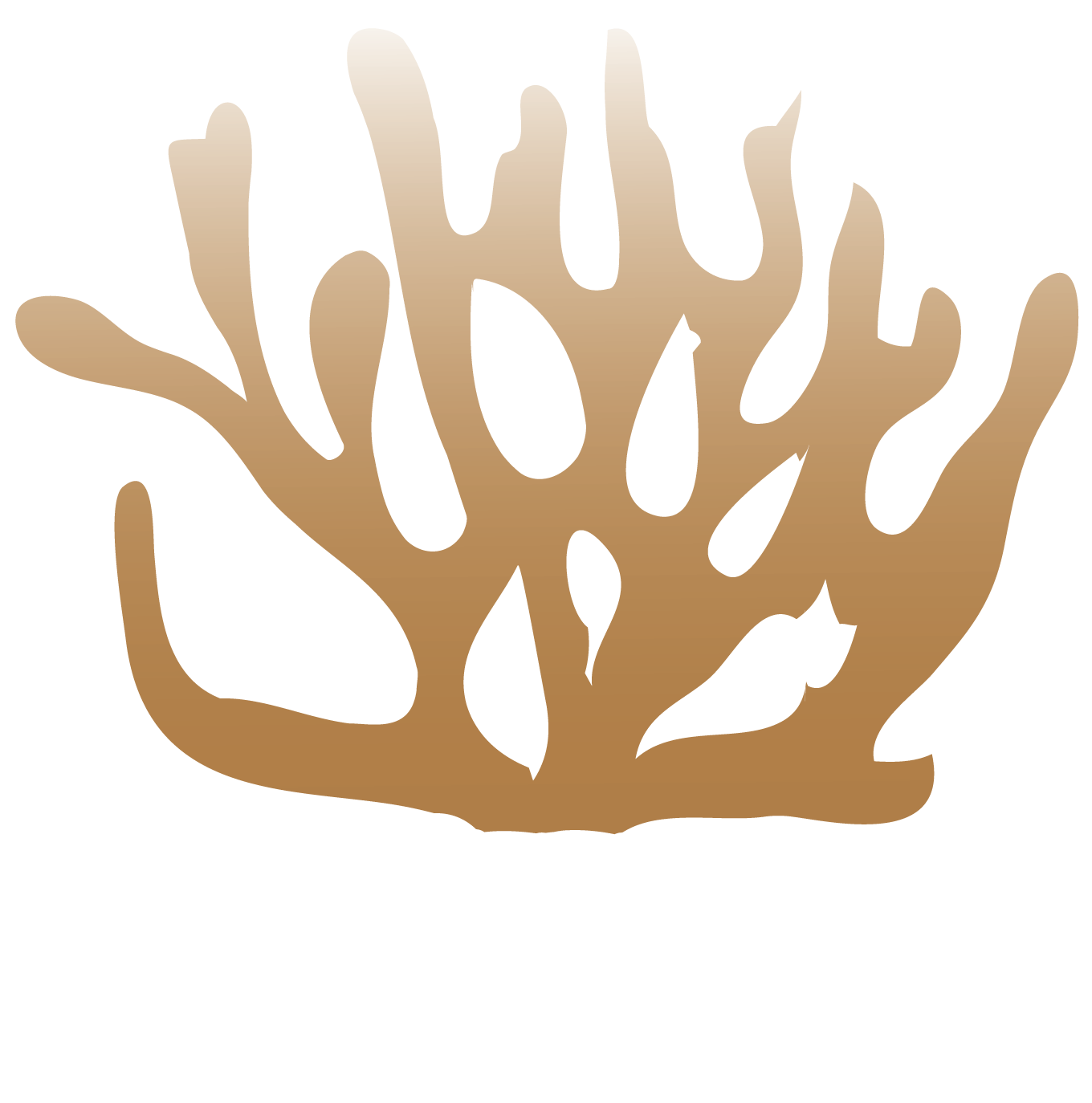 | **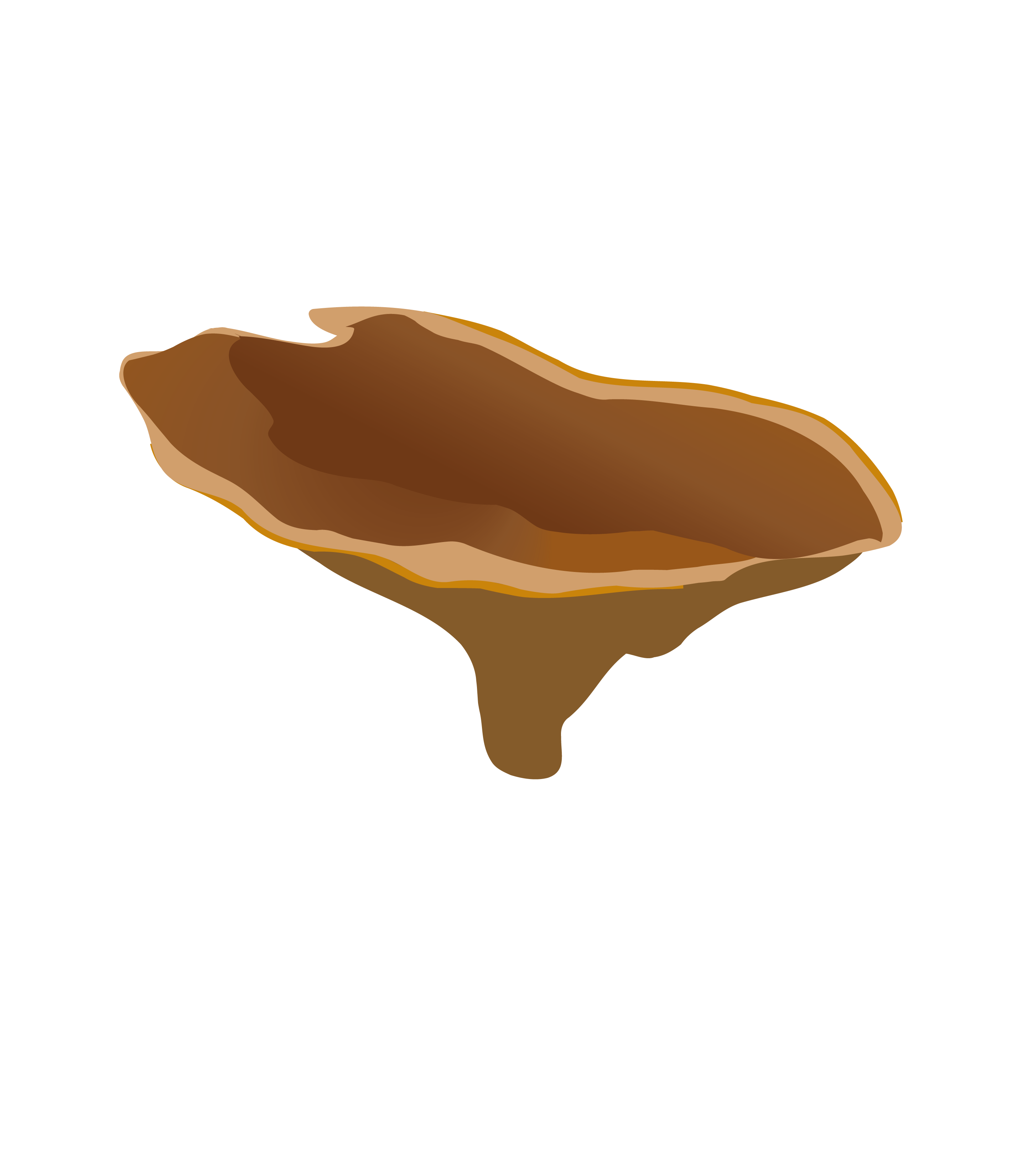** | **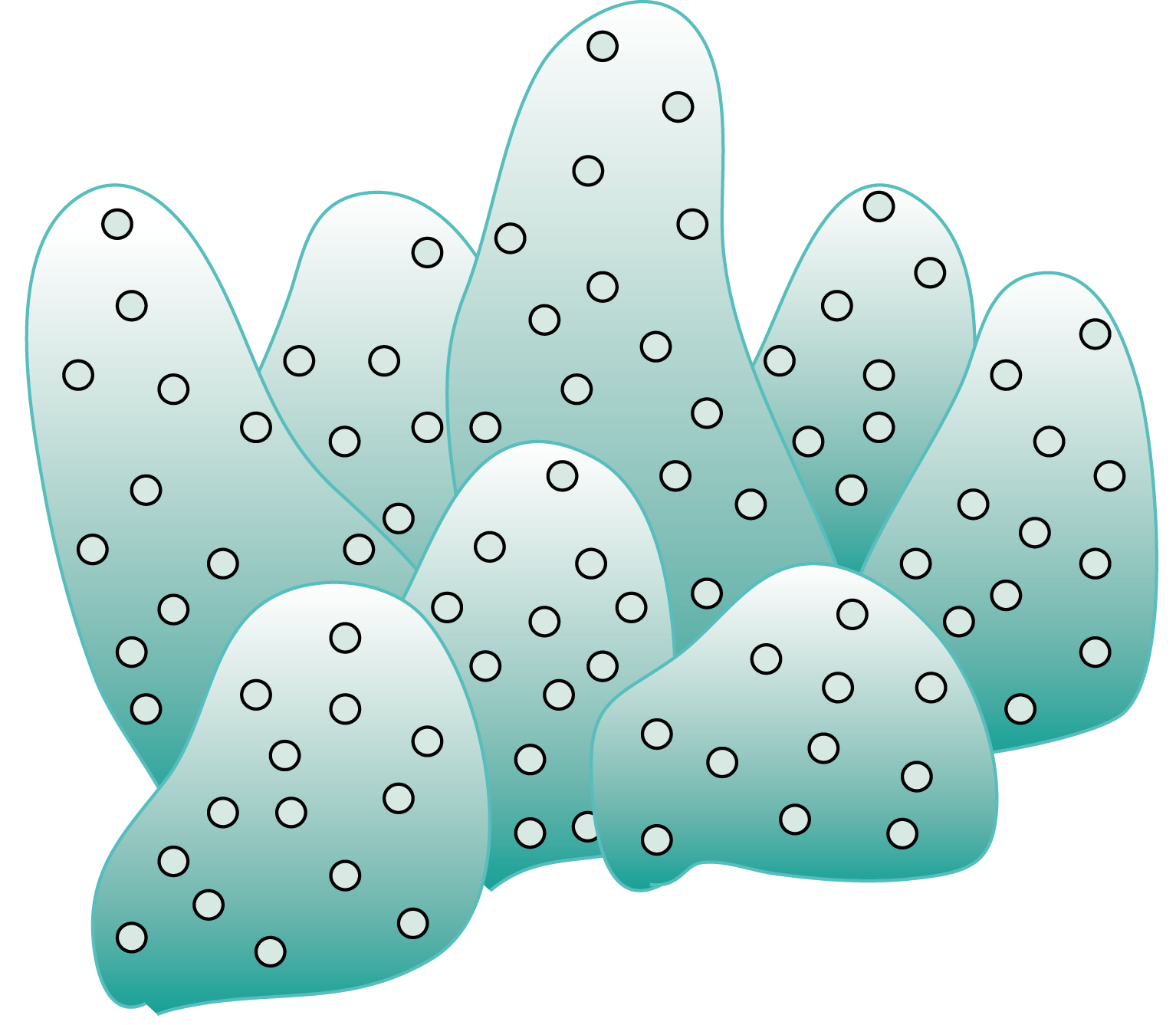** | 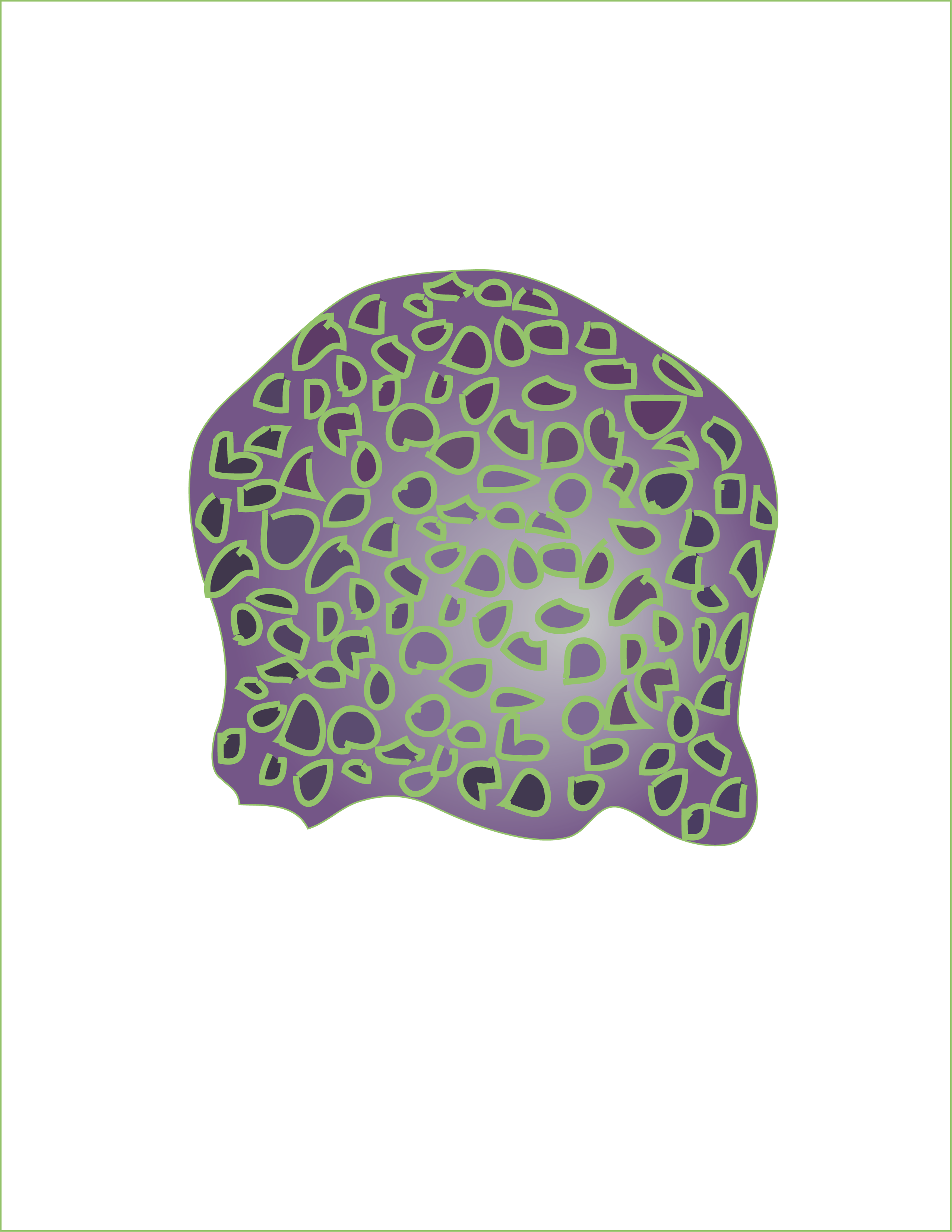 | 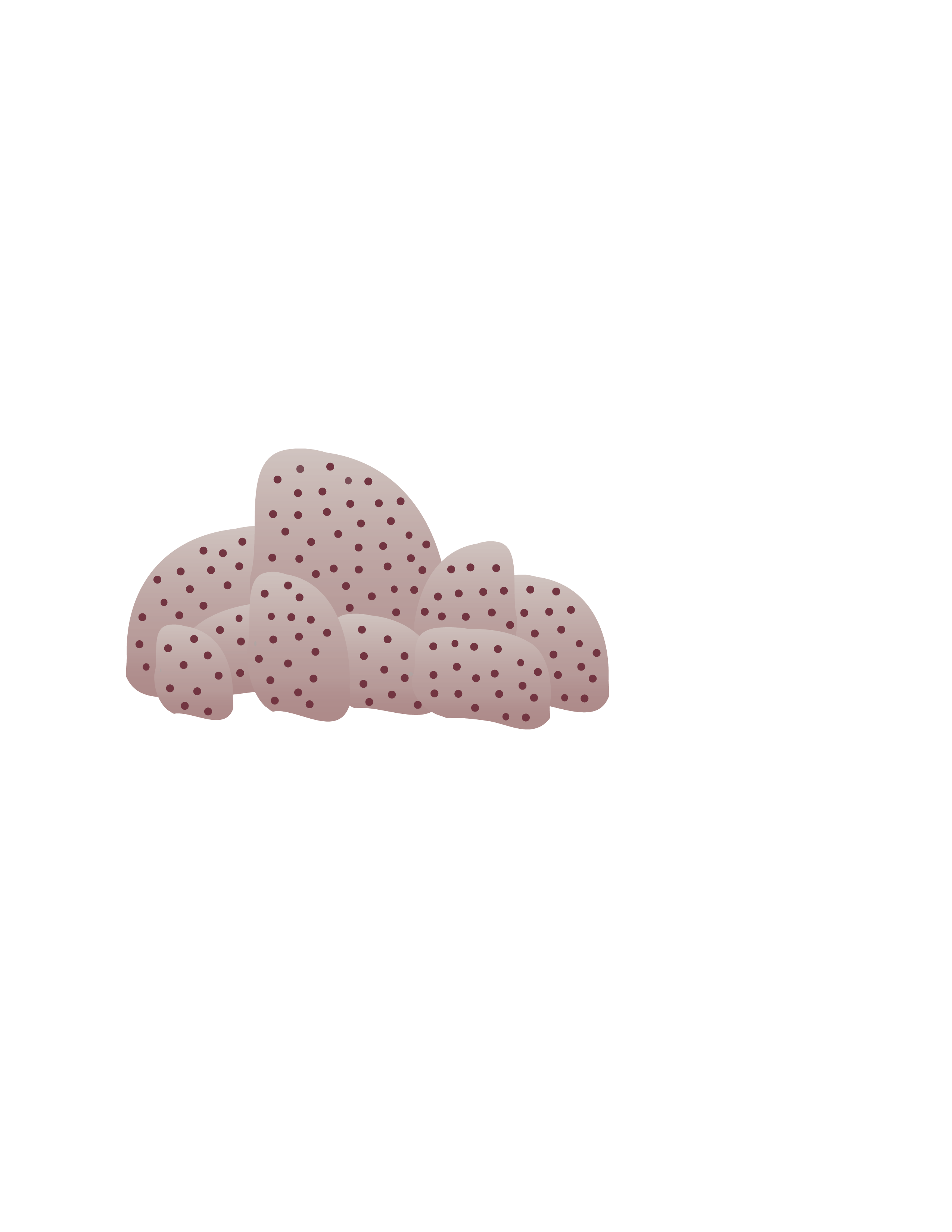 | **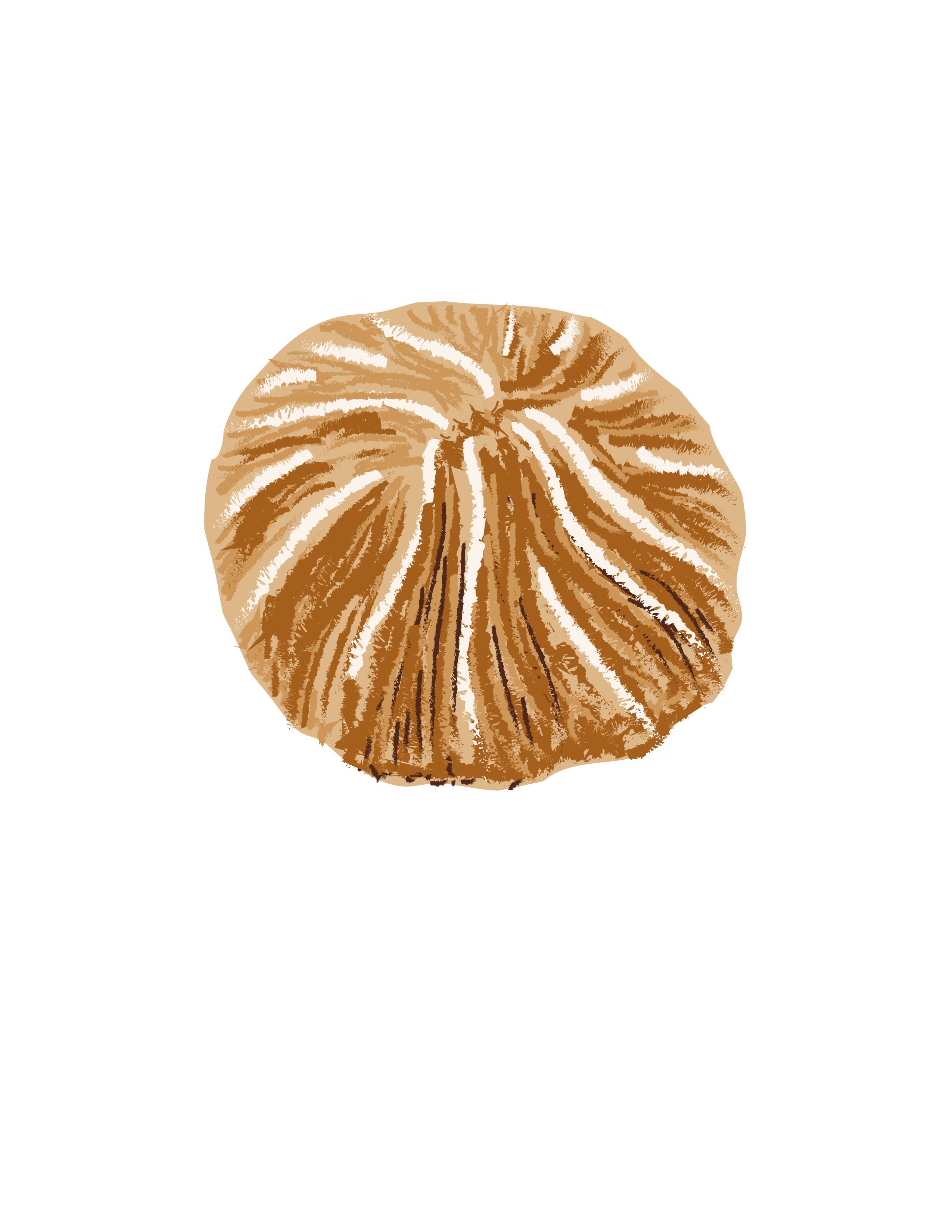** | **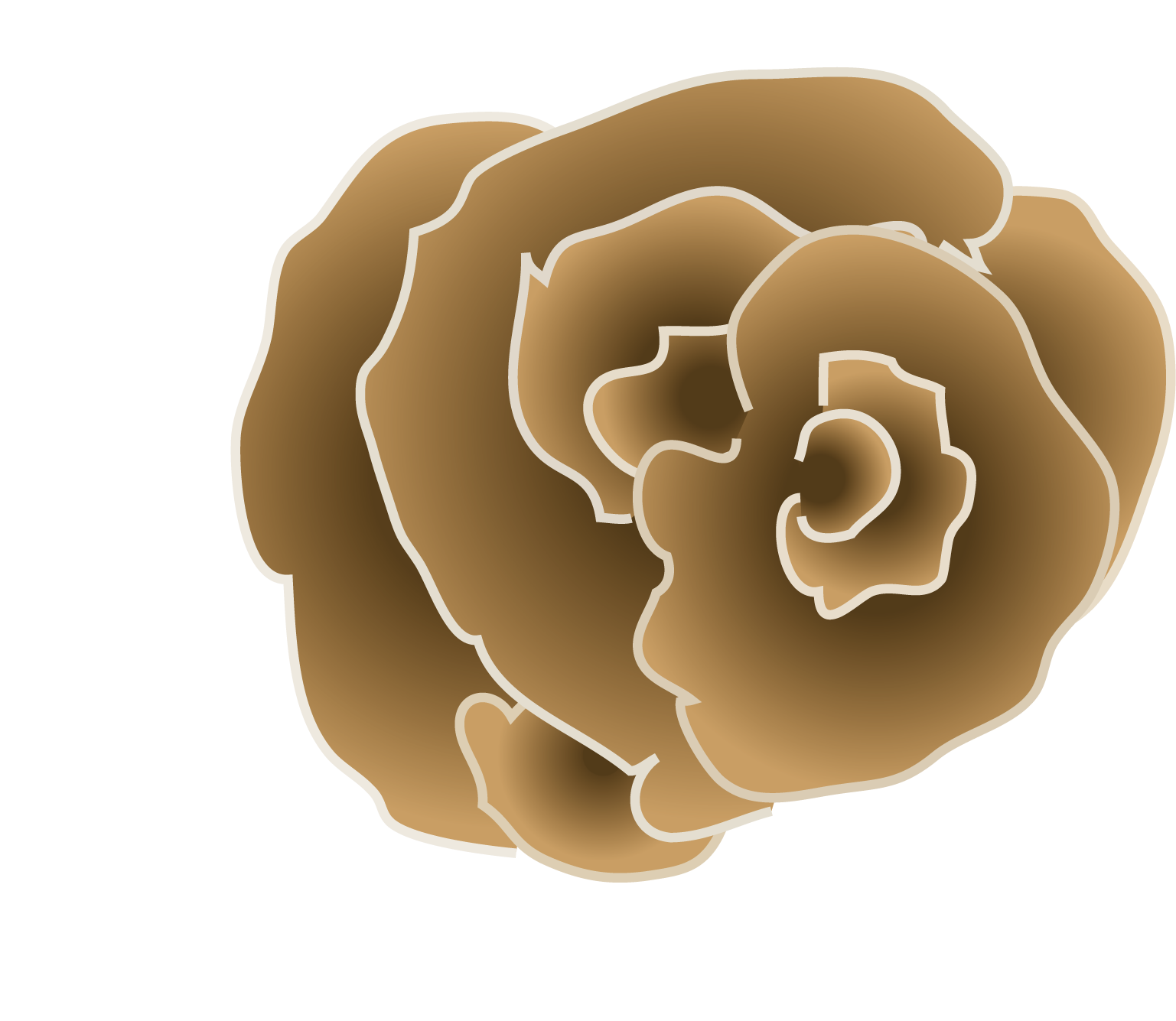** |
| *Acropora acuminata* | 1 | 1 | 1 | 0 | 0 | 0 | 0 | 0 |
| *Acropora aspera* | 1 | 1 | 0 | 0 | 0 | 0 | 0 | 0 |
| *Acropora cerealis* | 0 | 1 | 1 | 0 | 0 | 0 | 0 | 0 |
| *Acropora cytherea* | 0 | 0 | 1 | 0 | 0 | 0 | 0 | 0 |
| *Acropora digitifera* | 0 | 1 | 1 | 0 | 0 | 1 | 0 | 0 |
| *Acropora divaricata* | 0 | 1 | 1 | 0 | 0 | 0 | 0 | 0 |
| *Acropora elseyi* | 0 | 1 | 0 | 0 | 0 | 0 | 0 | 0 |
| *Acropora grandis* | 1 | 0 | 0 | 0 | 0 | 0 | 0 | 0 |
| *Acropora humilis* | 0 | 1 | 0 | 0 | 0 | 1 | 0 | 0 |
| *Acropora hyacinthus* | 0 | 0 | 1 | 0 | 0 | 0 | 0 | 0 |
| *Acropora intermedia*  *(= nobilis)* | 1 | 0 | 0 | 0 | 0 | 0 | 0 | 0 |
| *Acropora millepora* | 0 | 1 | 1 | 0 | 0 | 0 | 0 | 0 |
| *Acropora muricata (=formosa)* | 1 | 0 | 0 | 0 | 0 | 0 | 0 | 0 |
| *Acropora nasuta* | 0 | 1 | 1 | 0 | 0 | 0 | 0 | 0 |
| *Acropora pulchra* | 1 | 1 | 0 | 0 | 0 | 0 | 0 | 0 |
| *Acropora robusta* | 1 | 0 | 1 | 0 | 0 | 1 | 0 | 0 |
| *Acropora secale* | 0 | 1 | 1 | 0 | 0 | 0 | 0 | 0 |
| *Acropora subglabra* | 0 | 1 | 0 | 0 | 0 | 0 | 0 | 0 |
| *Acropora tenuis* | 0 | 1 | 1 | 0 | 0 | 0 | 0 | 0 |
| *Acropora valenciennesi* | 1 | 0 | 1 | 0 | 0 | 0 | 0 | 0 |
| *Acropora valida* | 0 | 1 | 1 | 0 | 0 | 0 | 0 | 0 |
| *Coeloseris mayeri* | 0 | 0 | 0 | 0 | 1 | 1 | 0 | 0 |
| *Cyphastrea serailia* | 0 | 0 | 0 | 1 | 1 | 1 | 0 | 0 |
| *Dipsastraea (=Favia) pallida* | 0 | 0 | 0 | 0 | 1 | 0 | 0 | 0 |
| *Dipsastraea (=Favia) speciosa* | 0 | 0 | 0 | 0 | 1 | 0 | 0 | 0 |
| *Favites abdita* | 0 | 0 | 0 | 0 | 1 | 1 | 0 | 0 |
| *Favites chinensis* | 0 | 0 | 0 | 0 | 1 | 1 | 0 | 0 |
| *Fungia concinna* | 0 | 0 | 0 | 0 | 1 | 0 | 1 | 0 |
| *Fungia fungites* | 0 | 0 | 0 | 0 | 1 | 0 | 1 | 0 |
| *Fungia paumotensis* | 0 | 0 | 0 | 0 | 1 | 0 | 1 | 0 |
| *Fungia scutaria* | 0 | 0 | 0 | 0 | 1 | 0 | 1 | 0 |
| *Galaxea fascicularis* | 0 | 0 | 0 | 0 | 1 | 1 | 0 | 0 |
| *Goniastrea pectinata* | 0 | 0 | 0 | 0 | 1 | 1 | 0 | 0 |
| *Goniastrea retiformis* | 0 | 0 | 0 | 1 | 1 | 1 | 0 | 0 |
| *Goniastrea* sp. | 0 | 0 | 0 | 1 | 1 | 1 | 0 | 0 |
| *Goniopora lobata* | 0 | 0 | 0 | 1 | 1 | 0 | 0 | 0 |
| *Helipora coerulea* | 0 | 1 | 0 | 1 | 1 | 1 | 0 | 1 |
| *Herpolitha limax* | 0 | 0 | 0 | 0 | 1 | 0 | 1 | 0 |
| *Hydnophora rigida* | 0 | 1 | 0 | 0 | 0 | 1 | 0 | 0 |
| *Isopora palifera* | 0 | 1 | 0 | 0 | 0 | 1 | 0 | 0 |
| *Isopora brueggemanni* | 1 | 1 | 0 | 0 | 0 | 0 | 0 | 0 |
| *Leptastrea pruinosa* | 0 | 0 | 0 | 0 | 0 | 1 | 0 | 0 |
| *Leptastrea purpurea* | 0 | 0 | 0 | 0 | 0 | 1 | 0 | 0 |
| *Lobophyllia corymbosa* | 0 | 0 | 0 | 0 | 1 | 0 | 0 | 0 |
| *Lobophyllia hemprichii* | 0 | 0 | 0 | 0 | 1 | 0 | 0 | 0 |
| *Millepora dichotoma* | 0 | 1 | 0 | 0 | 0 | 0 | 0 | 0 |
| *Millepora intricata* | 0 | 1 | 0 | 0 | 0 | 0 | 0 | 0 |
| *Millepora plathyphylla* | 0 | 0 | 0 | 0 | 1 | 1 | 0 | 0 |
| *Montipora digitata* | 0 | 1 | 0 | 0 | 0 | 0 | 0 | 0 |
| *Montipora foliosa* | 0 | 0 | 0 | 0 | 0 | 0 | 0 | 1 |
| *Montipora hispida* | 0 | 1 | 0 | 1 | 1 | 0 | 0 | 0 |
| *Montipora monasteriata* | 0 | 0 | 0 | 0 | 1 | 1 | 0 | 0 |
| *Montipora tuberculosa* | 0 | 0 | 0 | 0 | 1 | 1 | 0 | 0 |
| *Montipora verrucosa* | 0 | 0 | 0 | 1 | 1 | 1 | 0 | 0 |
| *Oulophyllia crispa* | 0 | 0 | 0 | 0 | 1 | 0 | 0 | 0 |
| *Pavona cactus* | 0 | 1 | 0 | 0 | 0 | 0 | 0 | 0 |
| *Pavona varians* | 0 | 0 | 0 | 0 | 1 | 1 | 0 | 1 |
| *Pavona venosa* | 0 | 0 | 0 | 0 | 1 | 1 | 0 | 0 |
| *Platygyra daedalea* | 0 | 0 | 0 | 0 | 1 | 1 | 0 | 0 |
| *Platygyra sinensis* | 0 | 0 | 0 | 0 | 1 | 1 | 0 | 0 |
| *Pocillopora damicornis* | 0 | 1 | 0 | 0 | 0 | 0 | 0 | 0 |
| *Pocillopora verrucosa* | 0 | 1 | 0 | 0 | 0 | 0 | 0 | 0 |
| *Porites cylindrica* | 0 | 1 | 0 | 0 | 0 | 0 | 0 | 0 |
| *Porites lichen* | 0 | 0 | 0 | 1 | 0 | 1 | 0 | 1 |
| *Porites lobata* | 0 | 0 | 0 | 0 | 1 | 0 | 0 | 0 |
| *Porites lutea* | 0 | 0 | 0 | 0 | 1 | 0 | 0 | 0 |
| *Porites nigrescens* | 0 | 1 | 0 | 0 | 0 | 1 | 0 | 0 |
| *Porites solida* | 0 | 0 | 0 | 0 | 1 | 0 | 0 | 0 |
| *Porites stephensoni* | 0 | 0 | 0 | 1 | 1 | 1 | 0 | 0 |
| *Symphyllia radians* | 0 | 0 | 0 | 0 | 1 | 0 | 0 | 0 |


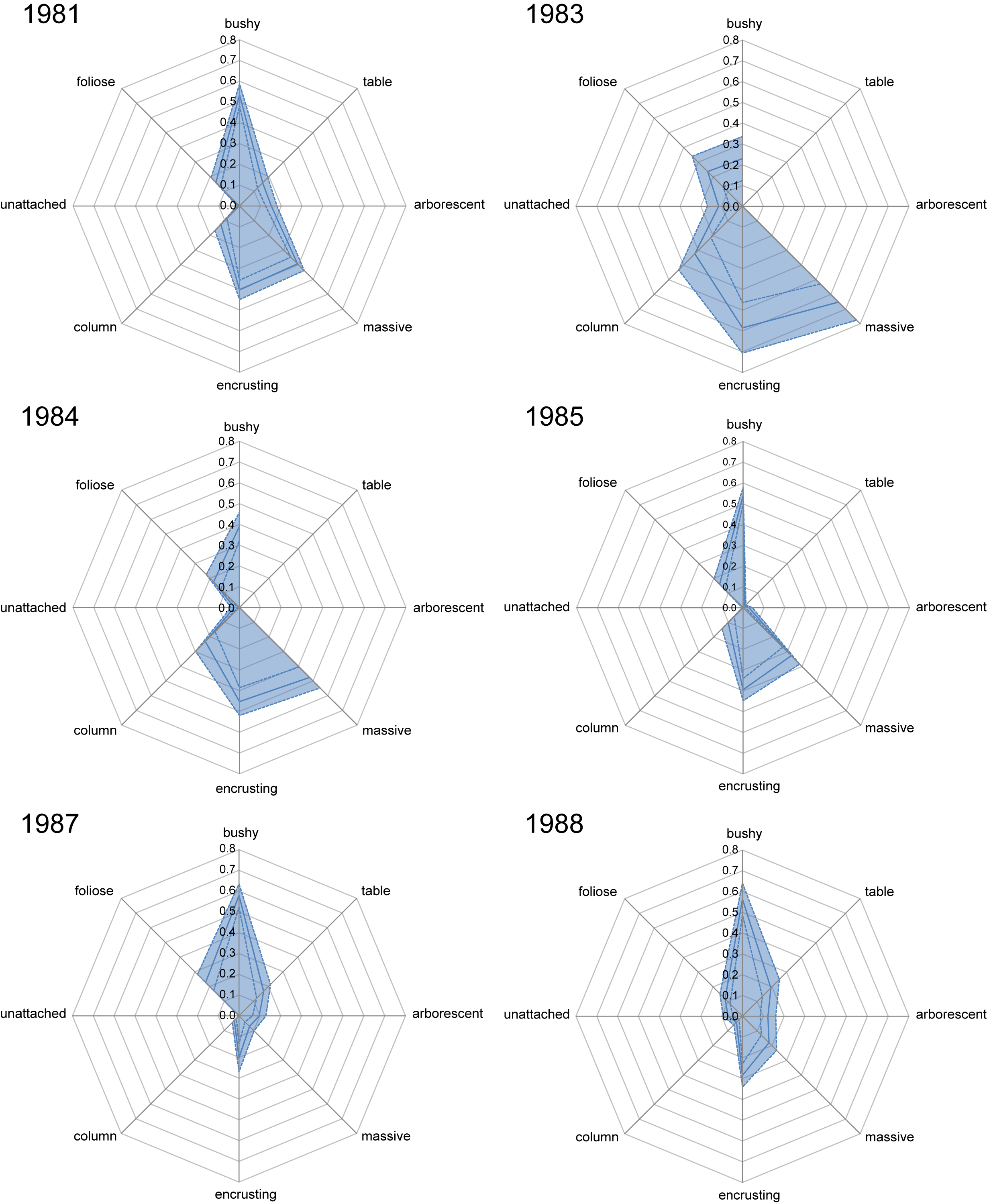


**Supplementary Figure S1.** **Yearly community-level weighted means (*CWM*) of morphological traits for each of the years surveyed.** Mean contribution (full line) ± standard error (dashed line). Change of the convex surface reflects a modification of the relative contribution of the different morphologies to the coral assemblage.


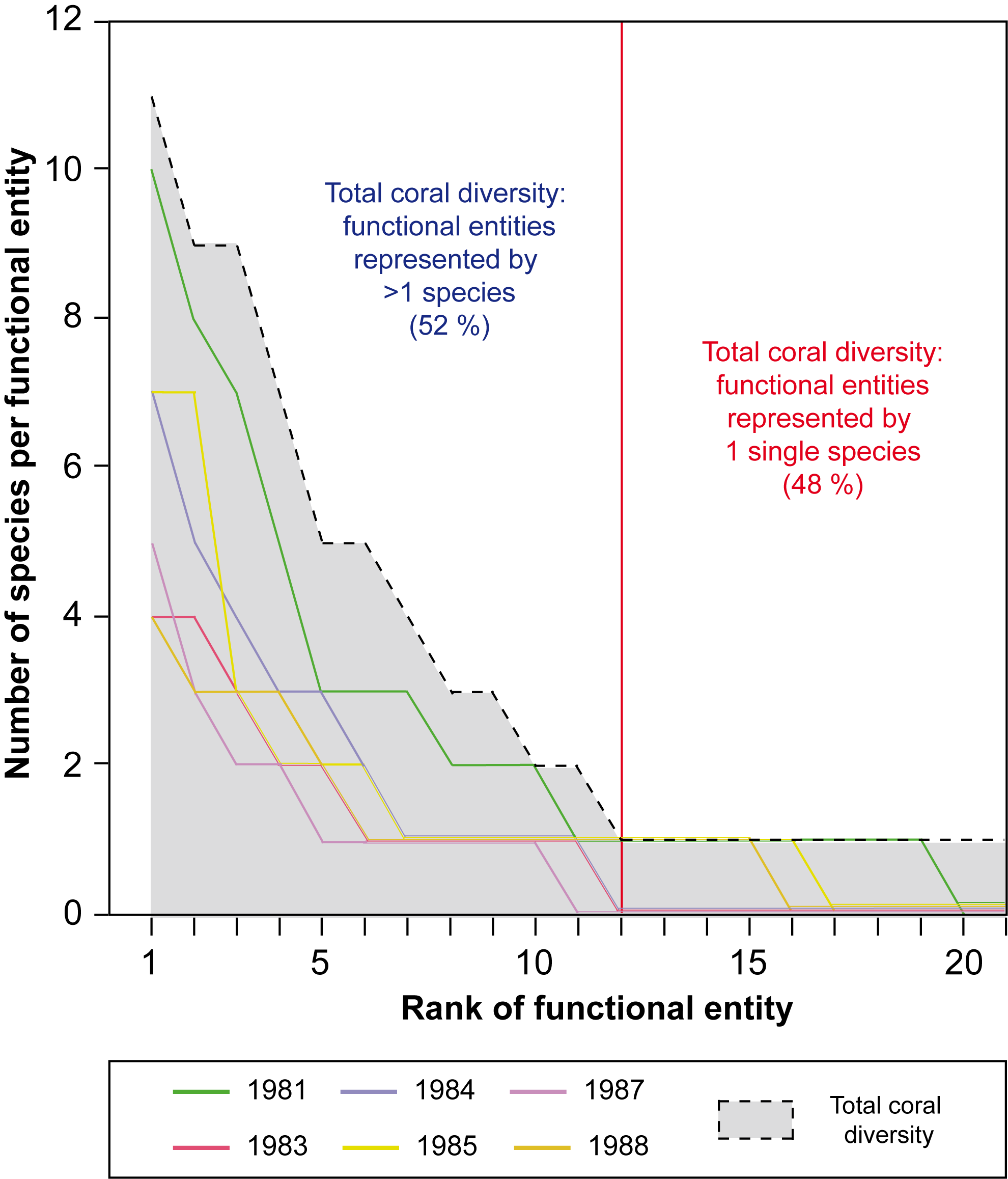


**Supplementary Figure S2.** **Coral species per functional entity.** The distribution of the number of coral species into functional entities is displayed for the total coral diversity recorded at Tikus Island and subsequent surveys (1981, 1983, 1984, 1985, 1987, and 1988).
