# Supplementary figures and images for: A functional approach to the structural complexity of coral assemblages based on colony morphological features
Source: Sci Rep. 2017 Aug 29;7:9849. doi: 10.1038/s41598-017-10334-w (PMC5575326; doi:10.1038/s41598-017-10334-w)

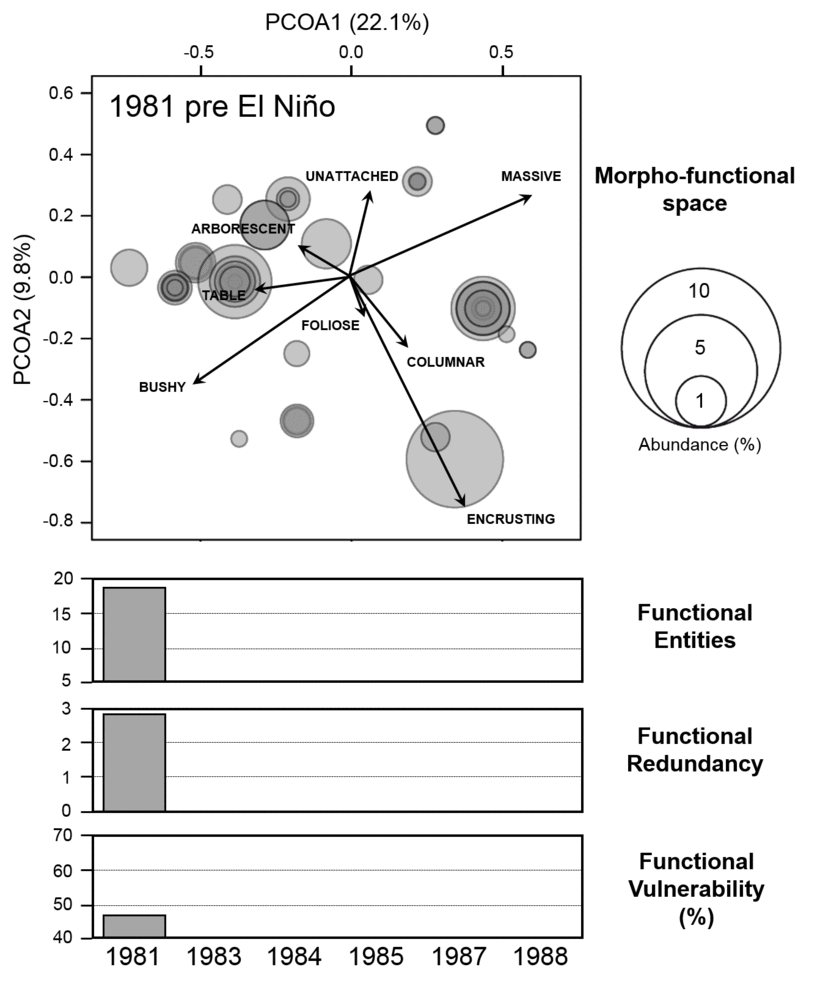

Supplement: Supplementary file 2 — Supplementary Video 1 [file 41598_2017_10334_MOESM2_ESM.gif]
